# Study on the Properties of Waterborne Epoxy Resin/Polyurethane Composite Modified Emulsified Asphalt

**DOI:** 10.3390/ma19112394

**Published:** 2026-06-04

**Authors:** Siyu Bo, Yitong Hou, Minda Ren

**Affiliations:** School of Civil Engineering, Inner Mongolia University of Technology, Hohhot 010051, China; 18447079197@163.com (S.B.);

**Keywords:** WEA, WER/PU, WER/PU-CMEA, flexibility, low-temperature crack resistance

## Abstract

Waterborne epoxy resin emulsified asphalt (WEA) is often used as a preventive maintenance material for asphalt pavement due to its excellent mechanical properties and high-temperature stability. However, its relatively poor toughness and low-temperature crack resistance limit its broader application. To address this issue, a PPG-IPDI-based waterborne polyurethane/epoxy resin (WER/PU) emulsion was synthesized via the prepolymer dispersion method using polypropylene glycol (PPG), isophorone diisocyanate (IPDI), 2,2-bis(hydroxymethyl)propionic acid (DMPA), and epoxy resin (E-44) as the main raw materials. Fourier transform infrared (FT-IR) spectroscopy confirmed that flexible polyurethane segments were successfully grafted onto the epoxy resin. This WER/PU emulsion was then incorporated as a modifier into emulsified asphalt to prepare waterborne polyurethane/epoxy resin composite-modified emulsified asphalt (WER/PU-CMEA). A series of laboratory tests were conducted to compare the compatibility, conventional properties, mechanical performance, high temperature rheological properties, low-temperature crack resistance and aging resistance of ordinary emulsified asphalt (OEA), WEA, and WER/PU-CMEA. Scanning electron microscopy (SEM) was employed to analyze the modification mechanism of WEA by WPU. The results show that WER/PU exhibits good compatibility with emulsified asphalt, and the synergistic effect of WER/PU significantly enhances the overall performance of the emulsified asphalt. Compared with WEA, WER/PU-CMEA shows a slight decrease in tensile strength and high-temperature stability, but it notably improves material compatibility, flexibility, bond strength, elongation at break, high-temperature creep-recovery performance, and low-temperature crack resistance. This study provides a promising approach for developing high-performance emulsified asphalt materials, which have strong application potential in pavement maintenance, waterproof coatings, and tack coats.

## 1. Introduction

As global transportation infrastructure continues to undergo construction and upgrades, it faces increasingly stringent challenges from heavy-duty traffic, high-density traffic flows, and extreme climatic conditions. In this context, the limitations of traditional petroleum-based asphalt pavement materials have become ever more apparent, particularly in terms of their long-term durability, resistance to high-temperature rutting, low-temperature crack resistance, and environmental compatibility [1]. Given these challenges, the development of new-generation pavement materials that offer high performance, extended service life, and environmental sustainability has emerged as both an urgent necessity and a pivotal research focus within the field of road engineering. Emulsified asphalt, with its benefits of simplified construction processes, energy efficiency, reduced carbon footprint, and applicability at ambient temperatures, is extensively utilized in preventive maintenance, cold recycling, and tack coat applications [2]. Nevertheless, its inherent shortcomings—such as relatively low strength, inadequate water resistance, and subpar performance at high temperatures—restrict its direct use in structural layers or scenarios with stringent demands. Consequently, enhancing the performance of emulsified asphalt through modification to achieve superior mechanical properties and road durability is a critical strategy for overcoming its application limitations and broadening its functional applications.

Epoxy asphalt, as a high-end composite material that significantly enhances asphalt performance through the formation of a three-dimensional thermosetting network structure via the reaction of epoxy resin with a curing agent, has been hailed as a “never-cracking” pavement material since its inception. It is renowned for its exceptional high-temperature stability, strong bonding strength, outstanding fatigue resistance, and excellent chemical corrosion resistance, demonstrating irreplaceable value in special and critical projects such as steel bridge deck paving, airport runways, and heavily loaded sections of highways [3]. However, traditional solvent-based epoxy asphalt systems are constrained by several issues, including high emissions of volatile organic compounds (VOCs), stringent construction safety requirements, sensitivity to substrate dryness, strict curing conditions, and relatively insufficient toughness, which limit their broader application [4]. In response to the call for green chemistry and sustainable development, waterborne epoxy resin (WER) systems have emerged. Using water as the dispersion medium, WER greatly reduces VOCs, improves the construction environment, and enhances adaptability to damp substrates [5]. The introduction of WER into emulsified asphalt to form waterborne epoxy resin modified emulsified asphalt (WEA) has opened a new pathway for road materials that combine high performance with environmental advantages [6]. Nevertheless, after curing, WER often imparts higher modulus and hardness to WEA, which may increase material brittleness and pose new challenges to flexibility and low-temperature crack resistance [7].

Meanwhile, waterborne polyurethane (WPU), as an environmentally friendly polymer material using water as the dispersion medium, demonstrates unique performance advantages due to the designability of its molecular structure [8]. By selecting different polyols, isocyanates, and chain extenders, the microphase-separated structure of soft and hard segments in the final product can be flexibly tailored, enabling a range of mechanical properties from high elasticity and high toughness to high hardness [9]. WPU typically exhibits excellent flexibility, wear resistance, impact resistance, adhesion, and good compatibility with various substrates [10]. Introducing WPU into the field of emulsified asphalt modification aims to utilize its flexible elastic network to improve the low-temperature performance, fatigue resistance, and potential self-healing capability of asphalt. However, asphalt modified solely with WPU may show limited improvement in high-temperature stability and strength [11].

It is evident that WER and WPU exhibit significant complementary properties—the former excels at constructing a rigid three-dimensional network, providing strength and stability, while the latter specializes in forming a flexible elastic phase, contributing to toughness and deformability. Inspired by the design concept of “combining rigidity with flexibility” in polymer materials, the synergistic use of both to modify emulsified asphalt—creating an organically hybridized, interpenetrating, or cooperatively cross-linked composite system—holds promise for achieving a modification effect where “the whole is greater than the sum of its parts”.

**Figure 1:**
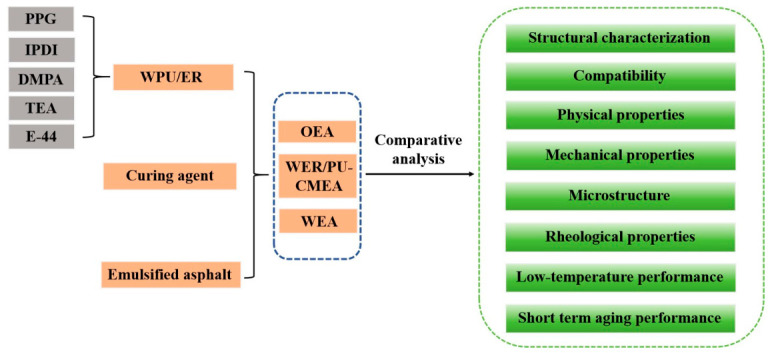
The roadmap for this study.

Based on this, to integrate the performance advantages of WER and WPU, this study synthesized a waterborne polyurethane/epoxy resin composite-modified emulsified asphalt (WER/PU-CMEA). Using the “prepolymer dispersion method”, polypropylene glycol (PPG) was selected as the soft segment, isophorone diisocyanate (IPDI) and 2,2-bis(hydroxymethyl)propionic acid (DMPA) served as the hard segment and hydrophilic chain extender, respectively, and epoxy resin (E-44) was introduced to participate in the reaction. Through chemical synthesis, polyurethane flexible chains were grafted onto the WER molecular chains, triethylamine (TEA) was used as an emulsifier to hydrate the epoxy resin, producing a structurally homogeneous composite emulsion (WER/PU). Subsequently, the WER/PU emulsion was used as a high-performance modifier to prepare WER/PU-CMEA. A comparative study was conducted on ordinary emulsified asphalt (OEA), waterborne epoxy emulsified asphalt (WEA), and WER/PU-CMEA in terms of structural characterization, compatibility, conventional physical properties, mechanical properties, microscopic morphology, high-temperature creep recovery performance, and low-temperature crack resistance. The study further elucidated the interaction mechanism and synergistic modification effects of WPU and WER within the asphalt system. The technical route of this research is illustrated in Figure 1.

## 2. Experimental Section

### 2.1. Raw Materials

The emulsified asphalt was purchased from Inner Mongolia Xinglu Emulsified Asphalt Plant, and its technical specifications are listed in Table 1. The waterborne epoxy resin (WER) emulsion was prepared in-house by emulsifying epoxy resin (E-44). The curing agent, primarily composed of triethylenetetramine (TETA), was sourced from Nantong Xingchen Synthetic Materials Co., Ltd. (Nantong, China). The technical specifications of the WER emulsion and the curing agent are provided in Table 2. Epoxy resin (E-44) was purchased from Dongguan Heima Chemical Co., Ltd. (Dongguan, China), with its technical specifications shown in Table 3. Polypropylene glycol (PPG), isophorone diisocyanate (IPDI), 2,2-bis (hydroxymethyl) propionic acid (DMPA), and triethylamine (TEA) were all obtained from Shanghai Aladdin Biochemical Technology Co., Ltd. (Shanghai, China), and their respective technical specifications are summarized in Table 4.

### 2.2. Preparation of WER/PU Emulsion

**Figure 2:**
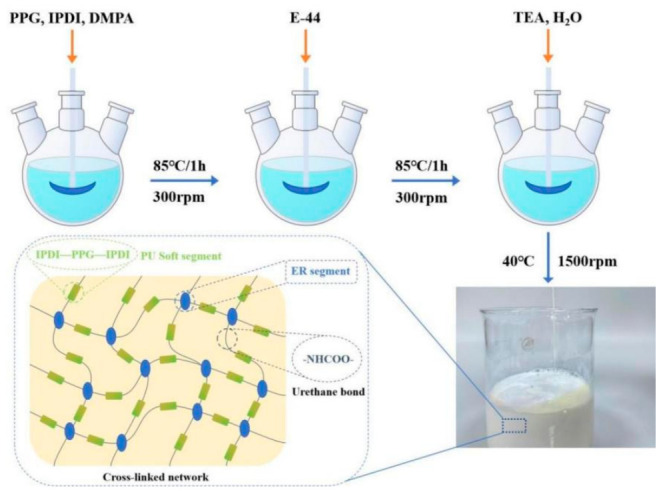
Preparation process of WER/PU emulsion.

Based on the “prepolymer dispersion method,” chemical modification was achieved through the reaction of epoxy resin E-44 with a polyurethane prepolymer. The specific preparation process is illustrated in Figure 2. In a dry three necked flask, 60 g of PPG, 12 g of IPDI, and 4 g of DMPA were sequentially added. After nitrogen protection, the mixture was placed in an 85 °C water bath and stirred at 300 rpm for 1 h to synthesize a prepolymer containing isocyanate groups (-NCO) and carboxyl hydrophilic groups at the end; Then, keeping the temperature and stirring speed constant, slowly add 18 g of epoxy resin E-44 to the prepolymer and continue the reaction for 1 h. Leverage the hydroxyl groups (-OH) present on the epoxy resin chain to react with residual isocyanate (-NCO) groups, maintaining a stoichiometric ratio of -NCO to -OH at 1:1. After the reaction, the system was cooled to 40 °C, 3 g TEA was added to neutralize the carboxyl group to weakly alkaline, and finally 90 g deionized water was slowly added under 1500 rpm high-speed shear for emulsification and dispersion. The proportion of total solid mass to water was controlled to make the final solid content reach 50%. After deaeration and filtration, a stable water-based epoxy resin/polyurethane (WER/PU) emulsion was prepared.

### 2.3. Preparation of WEA and WER/PU-CMEA

**Figure 3:**
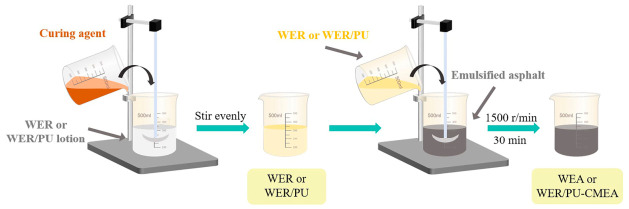
Preparation method of the WEA and WER/PU-CMEA.

The preparation processes of WEA and WER/PU-CMEA are illustrated in Figure 3. At room temperature, WER or WER/PU emulsion was uniformly mixed with a curing agent to obtain the corresponding composite material. This composite was then incorporated into emulsified asphalt at a mass ratio of 20% and subjected to high-speed shear mixing at 1500 r/min for 30 min, yielding either WEA or WER/PU-CMEA. To systematically compare and investigate the effects of WER and WER/PU on the performance of emulsified asphalt, ordinary emulsified asphalt (OEA) was used as a control group for parallel analysis.

### 2.4. Test Method

#### 2.4.1. Fourier Transform Infrared Spectroscopy (FT-IR)

Fourier transform infrared spectroscopy (FT-IR) was performed using an S5 spectrometer (Thermo Fisher Scientific, Waltham, MA, USA) to analyze changes in functional groups and characterize the structure of different emulsified asphalt samples. The test aimed to examine the grafting of WPU onto WER. Samples were prepared by the KBr pellet method, and the spectra were recorded over a wavenumber range of 4000 cm^−1^ to 400 cm^−1^.

#### 2.4.2. Storage Stability Test

**Figure 4:**
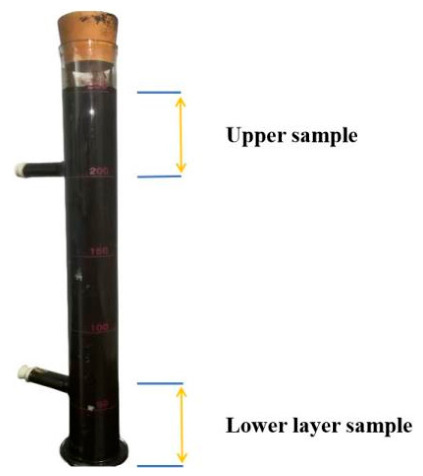
Stability test of emulsified asphalt storage.

According to Chinese industry standard JGJ 3410-2025 [12], different types of emulsified asphalt samples were slowly injected into stability test tubes until the liquid level reached the 250 mL mark on the tube wall. The tubes were then sealed with stoppers, as shown in Figure 4. After standing at room temperature for 5 d, the upper and lower sections of the samples were separately taken out, and their respective binder residue contents were measured as P_A_ (%) and P_B_ (%). The storage stability of the samples was calculated according to Equation (1) to evaluate the compatibility between WER, WER/PU, and the emulsified asphalt.

(1) $$S_{S}=\left|P_{A}-P_{B}\right|$$

where S_S_ indicates storage stability of the sample (%); P_A_ indicates binder residue content of the sample from the upper tube after storage (%); P_B_ indicates binder residue content of the sample from the lower tube after storage (%).

#### 2.4.3. Conventional Physical Performance Test

To investigate the effect of WER and WER/PU on the conventional physical properties of emulsified asphalt, the penetration, ductility (at 10 °C), and softening point of the binder residues of OEA, WEA, and WER/PU-CMEA were tested according to ASTM D5/D5M-13 [13], ASTM D113-17 [14], and ASTM D36/D36M-12 [15], respectively.

#### 2.4.4. Adhesive Strength Test

**Figure 5:**
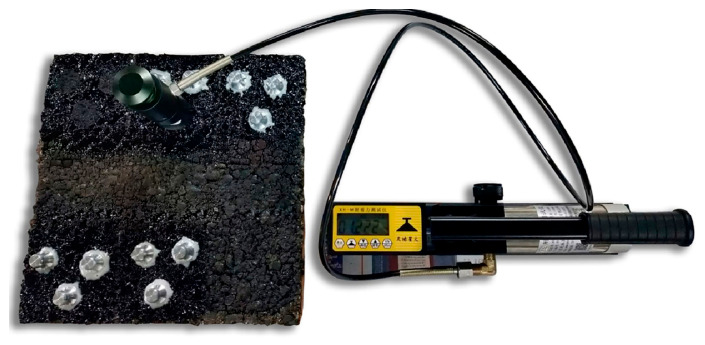
Pull-off test of emulsified asphalt.

In accordance with ASTM D4541-22 [16], different types of emulsified asphalt were uniformly applied to asphalt rutting slabs at a spreading rate of 0.4 kg/m^2^ and then left to cure at room temperature for 20 d. After curing, a pull-off stub was fixed to the center of the specimen surface using adhesive. Once the adhesive had fully set, the bond strength of the emulsified asphalt was measured using a portable adhesion tester (HCTC-10, Cuangzhou Zhulong Engineering Instrument Co., Ltd., Guangzhou, China) by performing a pull-off test on the stub at a constant displacement rate of 5 mm/min. The test procedure is illustrated in Figure 5.

#### 2.4.5. Tensile Strength Test

**Figure 6:**
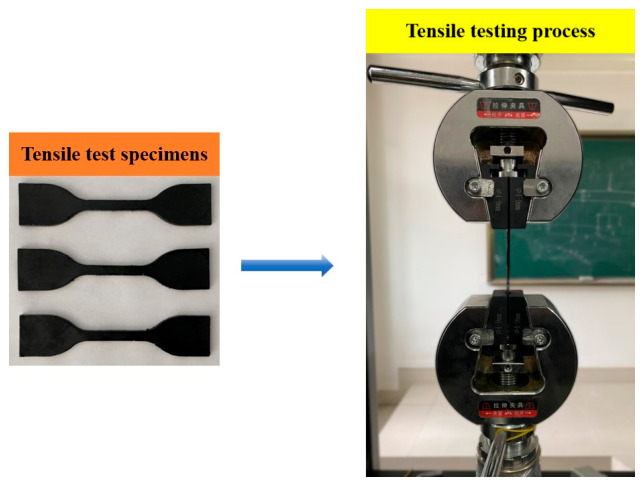
Tensile test of emulsified asphalt.

To investigate the mechanical properties of OEA, WEA, and WER/PU-CMEA binder residues, the tensile strength and elongation at break of different types of emulsified asphalt binder residues were tested according to ASTM D638-14 [17] using an electronic universal testing machine (CMT5105, MTS Systems Corporation, Eden Prairie, MN, USA). The tests were conducted at room temperature (25 °C) with a tensile speed of 500 mm/min. Dumbbell-shaped specimens were used, and the reported results represent the average of three repeated tests. The testing process is illustrated in Figure 6.

#### 2.4.6. Scanning Electron Microscope (SEM) Test

To observe the microstructure of the tensile fracture surfaces of different types of emulsified asphalt binder residues, the fractured tensile specimens were cut into cubes with a side length of 1 mm. The surfaces were then sputter-coated with gold on an aluminum plate to ensure clear observation of the microscopic morphology. The coated fracture samples were placed in a dedicated holder and examined using a scanning electron microscope (SIGMA 500, Carl Zeiss AG, Oberkochen, Germany) at a magnification of 1000 times.

#### 2.4.7. Dynamic Shear Rheological (DSR) Test

Using a TA Instruments DHR-2 dynamic shear rheometer, the WEA binder residues with different WER dosages were heated until they attained sufficient flowability. Subsequently, these residues were transferred into Polytetrafluoroethylene molds. Following thermal stabilization, the samples were subjected to rigorous rheological testing. In compliance with ASTM D7175-15 [18], The temperature scanning adopts a strain controlled load mode, with a parallel plate diameter of 25 mm and a gap set at 1 mm. Under sinusoidal oscillation load, the loading angular frequency is 10 rad/s, and the shear strain is set to 12%. The test temperature range is 46–72 °C; The frequency scanning test is conducted at a frequency of 0.01~10 Hz, using strain control mode with a controlled strain of 12%.

#### 2.4.8. Multi Stress Creep Recovery (MSCR) Test

According to ASTM D7405-20 [19], MSCR test was conducted on different types of emulsified asphalt binder residues using a rheometer (DHR-2, TA Instruments, New Castle, DE, USA) to investigate the influence of WER and WER/PU on the high-temperature creep recovery performance of asphalt. The test temperature was set at 60 °C. A 25 mm parallel plate geometry was used, and the test consisted of 10 cycles, with each cycle comprising 1 s of loading followed by 9 s of unloading. The stress levels applied were 0.1 kPa and 3.2 kPa, respectively.

#### 2.4.9. Bending Beam Rheology (BBR) Test

According to ASTM D6648-25a [20], BBR test was performed on different types of emulsified asphalt binder residues using a WXBBR-3Pro instrument (Hunan Wangxuan Technology Co., Ltd., Changsha, China). The test temperatures were set at −12 °C and −18 °C, respectively. The evaluation indices were creep stiffness modulus (S) and creep rate (m).

#### 2.4.10. Thin-Film Oven Test (TFOT)

According to ASTM D1754/D1754M-20, the binder residue of emulsified asphalt is subjected to short-term aging using a thin film oven test [21]. The test temperature was 163 °C and the heating time was 85 min. The anti-aging effect of emulsified asphalt was evaluated by measuring the difference in softening point and penetration ratio before and after aging.

## 3. Results and Analysis

### 3.1. Structural Characterization

**Figure 7:**
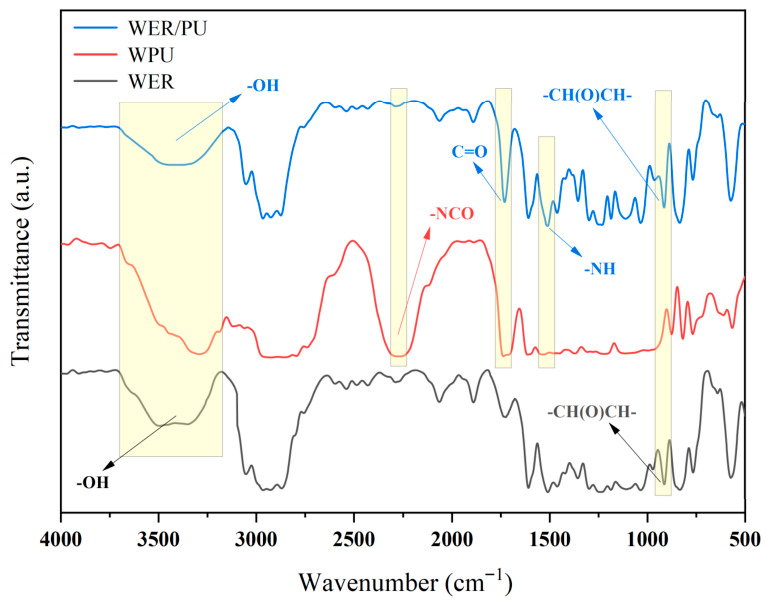
Infrared spectra of different types of emulsified asphalt.

The infrared spectra of WER, WPU, and WER/PU are shown in Figure 7. Within the range of 3500–3300 cm^−1^, a broad peak can be observed between WER and WER/PU, which is attributed to the -OH in the water and epoxy resin chains. At 2250 cm^−1^, the characteristic peak in WPU is caused by the -NCO functional group in isocyanates. The characteristic peaks at 1740 cm^−1^ and 1528 cm^−1^ correspond to the vibrations of C=O bonds and -NH in aromatic compounds. The absorption peak at 915 cm^−1^ represents the characteristic peak of epoxy groups [22]; From the characteristic peaks in the infrared spectrum, it can be seen that during the preparation of WER/PU, the characteristic peaks of the epoxy resin were retained, and the -OH in WER and—NCO in WPU were consumed, generating a large number of C=O bonds and -NH, indicating that the -OH on the epoxy resin chain reacted with the -NCO groups in WPU, thereby grafting polyurethane as a crosslinking point onto the main chain of the epoxy resin.

### 3.2. Compatibility Analysis

The storage stability of OEA, WEA, and WER/PU-CMEA was tested to analyze the compatibility of WER and WER/PU with emulsified asphalt. The test findings are presented in Table 5. As evident from the data in Table 5, OEA demonstrated superior storage stability and the highest degree of system compatibility. This exceptional performance can be primarily attributed to the emulsifier present in the emulsified asphalt, which functions as a surfactant. This surfactant features an amphiphilic molecular structure, with one end being asphalt-philic and the other hydrophilic. It precisely adsorbs at the interface between asphalt particles and the aqueous phase, substantially reducing interfacial tension and forming a strong interfacial protective film. Through mechanisms such as charge repulsion or steric hindrance, it effectively inhibits the collision and coalescence of asphalt particles, thereby ensuring the long-term stability of the system [23].

In contrast, WEA showed the poorest storage stability. This was due to the fact that asphalt was a non-polar or weakly polar hydrocarbon, while epoxy resin was a strongly polar polymer. The two were thermodynamically highly incompatible, with a strong tendency for phase separation, which led to a significant reduction in storage stability [24].

However, WER/PU-CMEA exhibited superior storage stability compared to WEA. Within this composite system, the molecular chains of WPU were notably flexible; its soft segments demonstrated a certain degree of compatibility with asphalt, while the hard segments displayed favorable compatibility or reactivity with the polar epoxy resin. More specifically, the soft segments of WPU were capable of more effectively penetrating or adsorbing onto the asphalt surface. Meanwhile, the -NCO/-OH groups within the WPU chains could engage in reactions with the epoxy or hydroxyl groups of the epoxy resin, leading to the formation of chemical bonds or robust hydrogen-bond interactions. The inclusion of WPU substantially enhanced the compatibility between asphalt and epoxy resin, thereby bolstering the mechanical strength and structural integrity of the interfacial layer [25]. Although this system was more complex than OEA, its storage stability was far superior to that of the plain waterborne epoxy-modified system, and its compatibility lay between that of OEA and WEA.

### 3.3. Conventional Physical Properties

**Figure 8:**
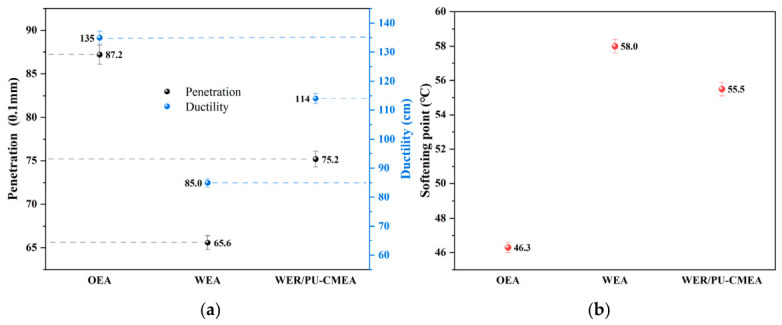
Conventional physical properties of different types of emulsified asphalt: (**a**) penetration and ductility; and (**b**) softening point.

Drawing on three standard physical indicators—penetration, softening point, and ductility—this study delved into the impacts of WER and WER/PU on the fundamental characteristics of emulsified asphalt binder residues. The outcomes are illustrated in Figure 8.

It was observed that, in comparison to unmodified OEA, WEA demonstrated a marked reduction in both penetration and ductility, while experiencing a substantial increase in the softening point. This phenomenon can be primarily ascribed to the fact that WER, as a thermosetting resin, forms a three-dimensional cross-linked network upon curing. This network effectively constrains the movement of asphalt molecules, bestowing the material with enhanced rigidity and high-temperature stability, albeit at the cost of some flexibility [26].

In contrast, the penetration, ductility, and softening point of WER/PU-CMEA all fell between those of OEA and WEA. This was mainly attributed to the introduction of flexible polyurethane molecular segments. The long polyurethane chains acted as a toughening agent, their incorporation diluted the cross-linking density of the rigid epoxy network to some extent, allowing the material to exhibit a certain elasticity under compression, hence showing lower hardness than pure WEA. Under tension, these chains could effectively absorb and dissipate energy, thereby granting the material greater deformability and ductility. Additionally, the introduction of polyurethane increased the free volume of molecular segments and enhanced their mobility, which also led to a corresponding decrease in the heat deflection temperature of the material [27].

### 3.4. Mechanical Properties

#### 3.4.1. Bond Strength

**Figure 9:**
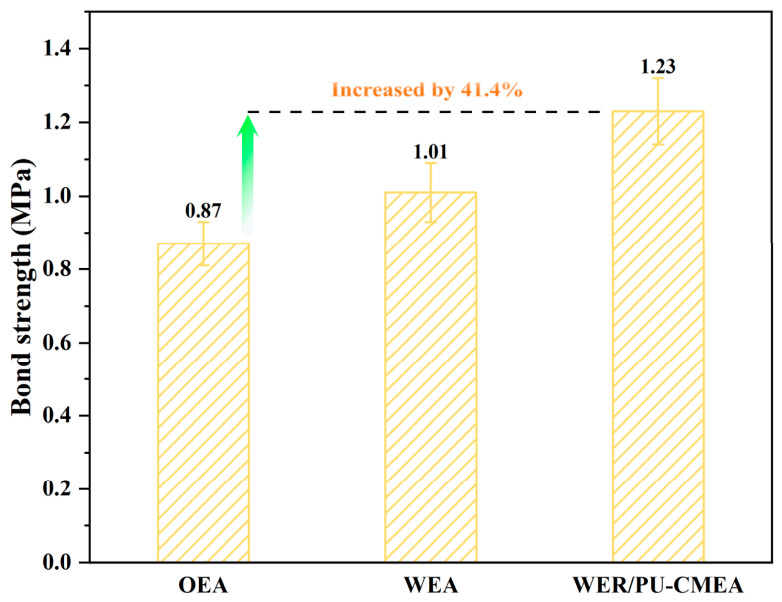
Bond strength of various emulsified asphalt.

Figure 9 presents the bond strength of various emulsified asphalt samples. As shown, the bond strength of OEA was only 0.87 MPa. This bonding force primarily relied on intermolecular interactions and micromechanical interlocking that developed after the emulsified asphalt cured upon contact with the substrate surface. The introduction of WER fundamentally changed the properties of emulsified asphalt. The dense three-dimensional cross-linked network formed after curing provided cohesive strength far exceeding that of conventional asphalt. When this network served as the main component of the bond layer, greater force was required to fracture it. Additionally, the active groups in the epoxy resin formed stronger physicochemical interactions with the contact surface, thereby enhancing the adhesion between WEA and the interface [28]. Consequently, the bond strength increased significantly to 1.01 MPa. The incorporation of WPU further improved the compatibility between WER and the emulsified asphalt, reducing internal micro-defects and enabling more uniform stress transfer. This allowed WER/PU-CMEA to undergo greater plastic deformation before failure, dissipating more energy [29]. As a result, the measured bond strength reached a peak value of 1.23 MPa, representing an increase of 41.4% compared to OEA.

#### 3.4.2. Tensile Strength

**Figure 10:**
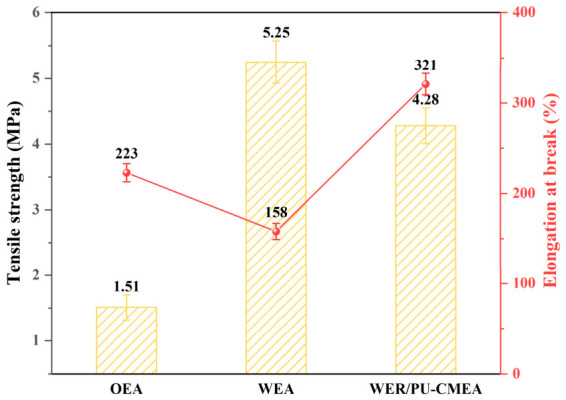
Tensile strength and elongation at break of each emulsified asphalt.

Figure 10 presents the tensile test results of binder residues from various emulsified asphalt samples. As shown, OEA exhibited a tensile strength of only 1.51 MPa and an elongation at break of 223%. This was because asphalt, as a thermoplastic material, derived its strength primarily from intermolecular van der Waals forces, making it susceptible to environmental influences. Under low-temperature or rapid loading conditions, asphalt behaved in a brittle manner, resulting in limited overall ductility. Moreover, asphalt consisted mainly of hydrocarbons and lacked a chemical cross-linked network, leading to easy slippage or fracture of molecular chains under stress [30].

In comparison, WEA showed a significant improvement in tensile strength, reaching 5.25 MPa, while its elongation at break decreased to 158%. This enhancement was attributed to the three-dimensional cross-linked network formed through the curing reaction between epoxy resin and asphalt, which substantially increased the material’s rigidity and strength. However, this cross-linked structure also restricted the mobility of molecular chains, thereby reducing the material’s deformability prior to fracture [31].

For WER/PU-CMEA, the tensile strength decreased slightly to 4.28 MPa compared to WEA, but its elongation at break rose markedly to 321%. This outcome resulted from the flexible polyurethane segments interpenetrating the epoxy network, which created a “rigid-flexible” composite structure that retained considerable strength while slightly compromising ultimate tensile values. The polyurethane segments imparted high elasticity and deformation capacity to the material, enabling greater energy absorption before failure. The synergistic interaction between the elastomeric characteristics of polyurethane and the epoxy network achieved an effective balance between strength and toughness [32]. According to Chinese standard GBT 30598-2014 [33], the tensile strength of epoxy asphalt materials should exceed 1.5 MPa, and the tensile strain should be greater than 200%. Therefore, WER/PU-CMEA met the required standards for use in epoxy asphalt paving on steel bridge decks.

#### 3.4.3. Microstructure of Fracture Surface

**Figure 11:**
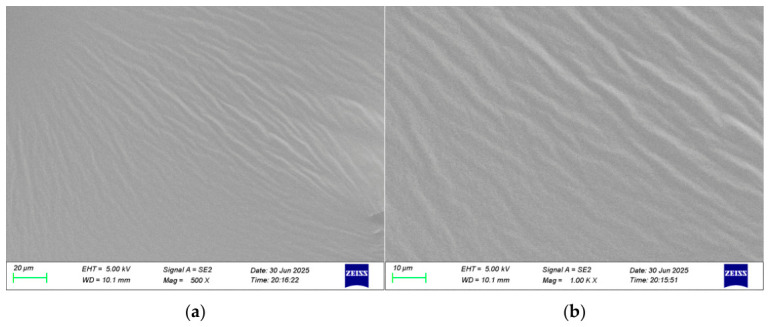

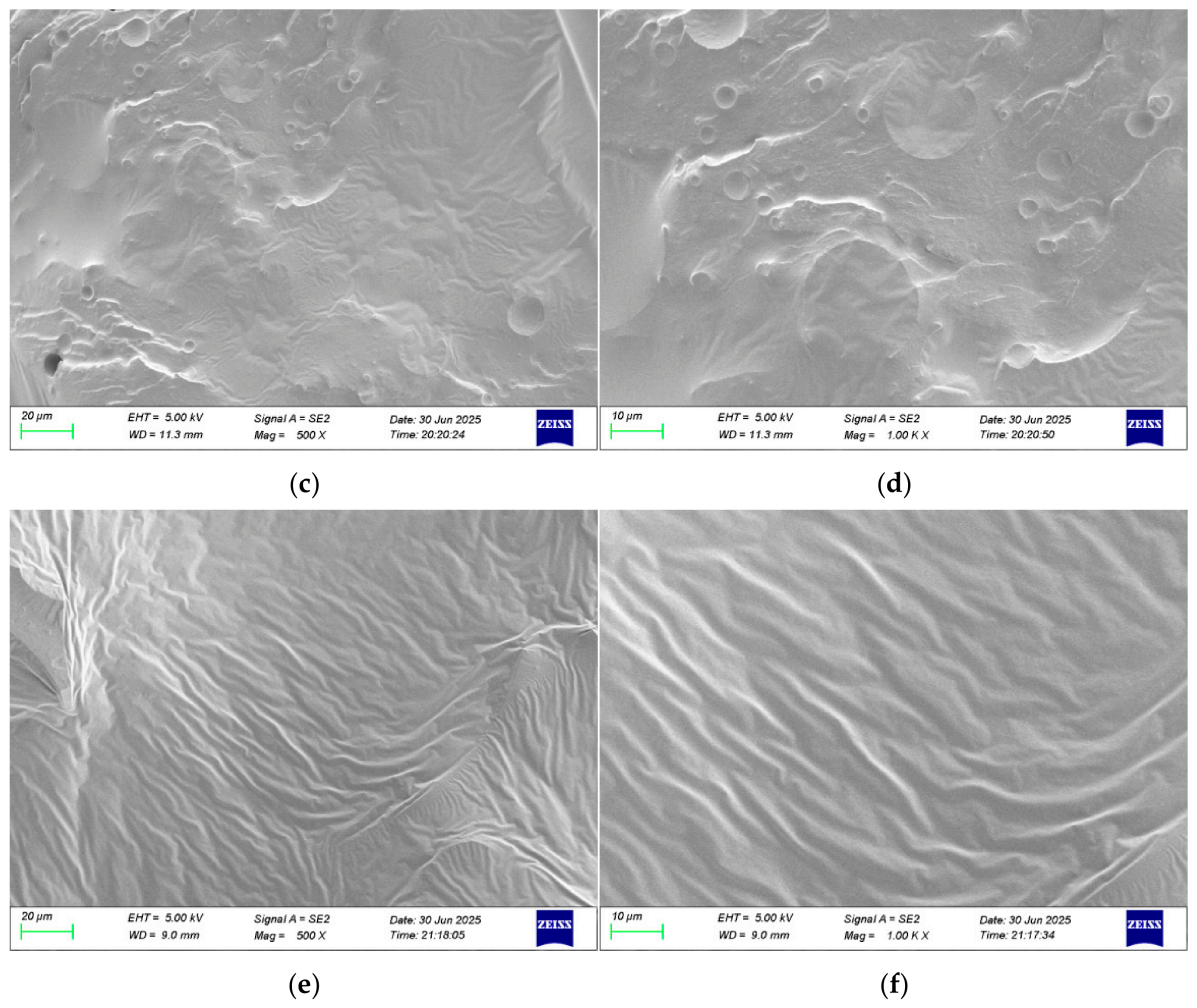
Microscopic morphology of asphalt tensile fracture surface: (**a**) OEA 500X; (**b**) OEA 1000X; (**c**) WEA 500X; (**d**) WEA 1000X; (**e**) WER/PU-CMEA 500X and (**f**) WER/PU-CMEA 1000X.

Figure 11 presents the microscopic morphology of the tensile fracture surfaces of binder residues from different types of emulsified asphalt. As shown in the figure, the fracture surface of OEA was relatively smooth and flat, with evident plastic flow and fibril formation, characteristic of ductile tearing [34]. In contrast, WEA exhibited a rough and uneven fracture surface with distinct phase interfaces, indicative of brittle fracture [35]. For WER/PU-CMEA, its fracture surface was rough, with many river-like patterns and numerous tough dimples, which was a sign of ductile fracture [36].

In summary, from OEA to WEA and then to WER/PU-CMEA, the fracture surfaces evolved from smooth plastic flow to rough brittle cleavage, and finally to a rough, dimple-dominated ductile morphology. This progression visually revealed the internal structural transformation from a homogeneous thermoplastic system to a rigid composite, and further to an interpenetrating network combining rigidity and flexibility. These microscopic observations provided clear evidence for the fundamental improvement in macroscopic mechanical properties.

### 3.5. High Temperature Rheological Properties

#### 3.5.1. Rutting Factor

**Figure 12:**
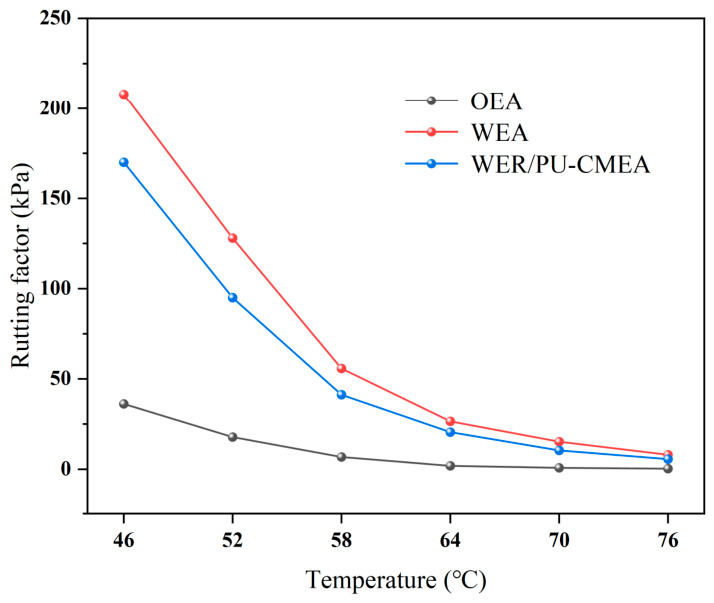
Changes in emulsified asphalt rutting coefficient under temperature scanning mode.

The rutting factor (G*/sinδ) is an important indicator for characterizing the high-temperature stability and rutting resistance of asphalt, and its numerical value directly reflects the ability of asphalt to resist deformation. The DSR temperature scanning test shows that, as shown in Figure 12, WEA exhibited the highest rutting factor under the same temperature conditions. Particularly at 76 °C, the rutting factor of WEA increased by 150% and 42.9% compared to OEA and WER/PU-CMEA, respectively. This indicated that WER could significantly enhance the high-temperature performance of asphalt. This improvement could primarily be attributed to the thermosetting properties of WER. After the emulsified asphalt cured, the rigid framework structure established by WER enhanced its resistance to high-temperature deformation. Additionally, WPU formed a partially interpenetrating network with the epoxy resin, resulting in a lower crosslinking density compared to a pure epoxy system, which consequently weakened the deformation resistance of the emulsified asphalt under high-temperature conditions.

#### 3.5.2. Complex Modulus (G*)

**Figure 13:**
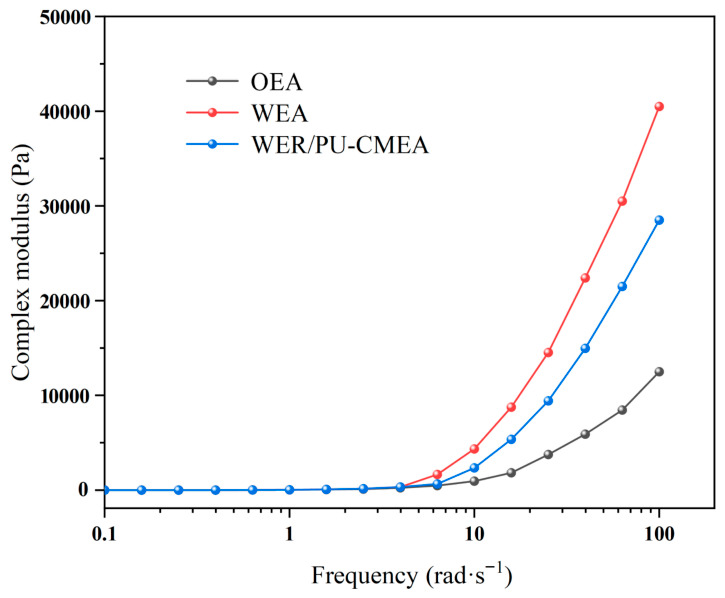
Complex modulus variation in emulsified asphalt in frequency scanning mode.

As a multiphase polymer composite material system, the mechanical behavior and deformation characteristics of WER/PU-CMEA are significantly dependent on the frequency characteristics of external loads. Figure 13 shows the regularity of the variation in viscoelastic parameters of different types of emulsified asphalt with frequency at an ambient temperature of 60 °C. It can be seen that in the low-frequency loading region, the modified emulsified asphalt exhibited a relatively low complex modulus G* value. However, when the loading frequency exceeded 10 rad/s and continued to rise, G* showed a significant exponential growth trend. The modification effects of WER and WER/PU on G* were mainly reflected in the high-frequency loading region. By comparing the frequency modulus curves of different types of emulsified asphalt under various temperature conditions, it was found that, under the same frequency conditions, the G* value of WEA was the highest. This phenomenon indicated that WER could effectively enhance the load resistance of asphalt, while the flexible component of WPU weakened the load resistance of asphalt.

#### 3.5.3. Creep Recovery Performance

In asphalt pavement, rutting primarily results from the continuous accumulation of residual deformation. To meet various performance requirements, asphalt binder must possess both excellent resistance to deformation and effective creep recovery capability. In light of this, the multiple stress creep recovery (MSCR) test is widely used to evaluate the deformation recovery characteristics of asphalt materials under high-temperature conditions, owing to its distinctive advantages.

**Figure 14:**
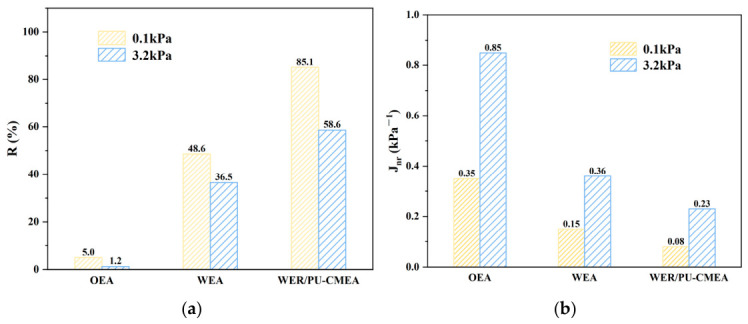
MSCR test results of each emulsified asphalt: (**a**) R and (**b**) J_nr_.

Figure 14 presents the creep recovery rate (R) and non-recoverable creep compliance (J_nr_) of asphalt samples tested at 60 °C under stresses of 0.1 kPa and 3.2 kPa, respectively. Here, R refers to the percentage of recoverable deformation relative to the total deformation after unloading. A higher R value indicates a greater proportion of elastic components in the material and stronger resistance to permanent deformation. J_nr_, on the other hand, reflects the permanent deformation per unit stress after a loading–unloading cycle. A lower Jnr value signifies better resistance to permanent deformation [37].

The test results show that as the stress increased from 0.1 kPa to 3.2 kPa at 60 °C, all emulsified asphalt specimens exhibited a decrease in R and an increase in J_nr_. This indicated that higher stress levels had a more pronounced impact on the asphalt system, making emulsified asphalt mixtures more susceptible to rutting. OEA demonstrated the lowest R and the highest J_nr_, representing the poorest creep recovery performance. This was because asphalt was a typical viscoelastic fluid whose deformation mainly stemmed from irreversible molecular chain slippage. At high temperatures or under sustained loading, the elastic recovery component was minimal, with most deformation converting into permanent plastic flow, which readily led to rutting.

Compared with OEA, WEA showed a significant increase in R and a notable decrease in J_nr_, indicating better creep recovery performance. This improvement was attributed to the three-dimensional dense cross-linked network formed by the reaction between epoxy resin and asphalt, which effectively “locked” asphalt molecules within the structure. This network exhibited high elasticity and could almost fully recover after stress removal, contributing to excellent rutting and flow resistance [31].

Compared with WEA, WER/PU-CMEA showed further improvement in creep recovery performance. This was due to the introduction of flexible polyurethane segments into the epoxy network, which increased the elastomeric component within the rigid epoxy matrix. While the rigid epoxy network resisted deformation, the flexible polyurethane network acted like “molecular springs”, absorbing and storing energy. Upon stress removal, the entropy-driven recoil of the polyurethane chains drove the entire material back to its original shape [38].

### 3.6. Low Temperature Crack Resistance

**Figure 15:**
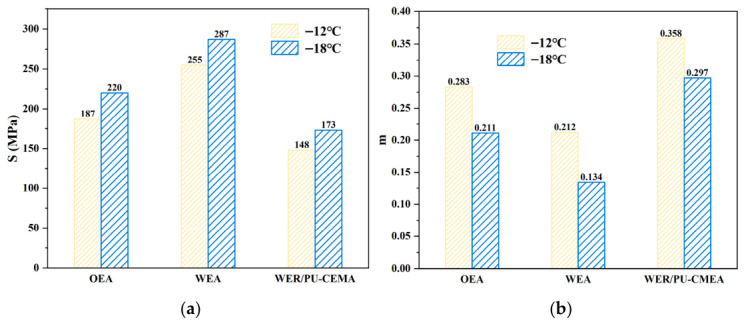
BBR test results of each emulsified asphalt: (**a**) S and (**b**) m.

Figure 15 presents the BBR test results of various emulsified asphalt samples. Analysis of the experimental data indicates that the creep characteristic parameters of the asphalt specimens, creep stiffness (S) and creep rate (m), exhibit a systematic correlation with temperature variation. Specifically, creep stiffness (S) shows a positive correlation with temperature, whereas creep rate (m) demonstrates a negative correlation with temperature. The incorporation of WER reduced the low-temperature crack resistance of the emulsified asphalt. This was because, in WEA, epoxy resin and curing agents underwent chemical reactions with asphalt, forming a highly dense three-dimensional cross-linked network [31]. This structure imparted excellent high-temperature stability and strength, but it also severely restricted the movement of molecular chains at low temperatures, making the material stiff and lacking flexibility. As a result, stress could not be effectively released, leading to pronounced low-temperature brittleness. In contrast, WER/PU-CMEA exhibited superior low-temperature crack resistance. In this system, polyurethane engaged in ring-opening polymerization with epoxy resin, introducing a large number of flexible long-chain segments into the rigid three-dimensional epoxy network. These flexible chains acted like “springs”, allowing the material to undergo a certain degree of deformation and stress relaxation even at low temperatures, thereby effectively resisting cracking [31,38].

### 3.7. Short Term Aging Performance

To evaluate the aging resistance of emulsified asphalt residue, the softening point difference and penetration ratio can be evaluated. The softening point difference refers to the softening point difference before and after the asphalt aging test. The smaller the value is, the better the aging resistance of the asphalt. The penetration ratio refers to the ratio of the asphalt penetration after the aging test to that before the aging test. The higher the percentage of residual penetration is, the stronger the anti-aging ability of the asphalt and the more favorable the pavement performance. The TFOT results of different types of emulsified asphalt are shown in Table 6.

Table 6 shows that the addition of WER and WER/PU improves the anti-aging performance of emulsified asphalt. Specifically, after adding WER, the softening point difference in emulsified asphalt decreased from 6.7 °C to 5.2 °C, and the penetration rate increased from 58.8% to 62.3%. After adding WER/PU, the softening point difference in emulsified asphalt decreased from 6.7 °C to 4.6 °C, and the penetration rate increased from 58.8% to 75.7%. This was due to the addition of light components of aging asphalt after WER/PU curing, the dissolution of asphaltene produced in the aging process, the reduction in the relative content of asphaltene, and the improvement of the anti-aging performance of asphalt.

## 4. Conclusions

Based on the “prepolymer dispersion method,” this study developed a waterborne polyurethane/epoxy resin composite modified emulsified asphalt (WER/PU-CMEA). Its compatibility, conventional physical properties, mechanical performance, microstructure, high temperature rheological properties, low-temperature crack resistance and aging resistance were compared and analyzed with those of ordinary emulsified asphalt (OEA) and waterborne epoxy resin modified emulsified asphalt (WEA). The main findings are as follows:

(1) The infrared spectra of each emulsified asphalt indicate that the spectrum of WER/PU CMEA retained the characteristic absorption peaks of OEA and WEA, while also exhibiting the characteristic peaks of PU, indicating that the PU segments had been successfully grafted onto the epoxy resin during the preparation process of WER/PU CMEA.
(2) The introduction of WPU significantly improved the compatibility between emulsified asphalt and WER, enhanced the mechanical strength and structural integrity of the interfacial layer, and resulted in better storage stability of WER/PU-CMEA compared to WEA.
(3) In WER/PU CMEA, the incorporation of polyurethane diluted the crosslink density of the epoxy rigid network to a certain extent, causing the material to exhibit some elasticity under compression, resulting in lower hardness than pure WEA; The introduction of polyurethane increased the free volume of molecular segments, which led to a corresponding decrease in the material’s thermal deformation temperature.
(4) The incorporation of WPU enhanced the adhesive properties of WEA. Although it led to a slight decrease in tensile strength, the elongation at break was significantly improved, reaching up to 321%. Microscopic examination of the fractured surface of the tensile specimens revealed that WER/PU-CMEA possessed a hybrid network structure combining rigidity and flexibility, which resulted in a ductile fracture mode.
(5) The MSCR and BBR test results demonstrated that in WER/PU-CMEA, the ring-opening polymerization between polyurethane and epoxy resin introduced a substantial number of flexible long-chain segments into the rigid three-dimensional epoxy network. This structure endowed WER/PU-CMEA with superior high-temperature creep recovery and low-temperature crack resistance compared to both OEA and WEA.
(6) As the light component of aging asphalt is added after WER/PU curing, the asphaltene produced during aging is dissolved, the relative content of asphaltene is reduced, and the aging resistance of asphalt is improved.

## Figures and Tables

**Table 1:** Technical parameters of emulsified asphalt.

Technical Parameters		Test Index
Properties of Binder Residue	Solid content/%	58.5
	Penetration (25 °C)/0.1 mm	87.2
	Softening point/°C	46.3
	Ductility (25 °C)/cm	>150

**Table 2:** Main technical indexes of WER emulsion and curing agent.

Material	Appearance	Solid Content (%)	Epoxy Value (eq/100 g)	Particle Size (μm)	Active Hydrogen Equivalent (g/eq)	PH
WER emulsion	milky liquid	50	0.23	<2.0	-	7.3
Curing agent	pale yellow liquid	50	-	-	220	8.5

**Table 3:** Main technical indicators of E-44.

Appearance	Epoxy Value (eq/100 g)	Viscosity (mPa·s, 25 °C)	Organic Chlorine Value (eq/100 g)	Volatile Matter (%)
Light yellow viscous liquid	0.46	30,000	<0.014	<1.0

**Table 4:** Main technical indicators of PPG, IPDI, DMPA and TEA.

Material	Appearance	Molecular Weight (g/mol)	Hydroxyl Value (mgKOH/g)	NCO Content (%)	Acid Value (mgKOH/g)	Density (g/cm^3^)
PPG	Colorless liquid	2000	56	-	<0.5	1.00
IPDI	Slightly yellow liquid	222	-	37.8	-	1.05
DMPA	white powder	134	830	-	414	-
TEA	pale yellow liquid	149	-	-	-	1.12

**Table 5:** Storage stability of different types of emulsified asphalt.

Material	Upper Solid Content (%)	Lower Solid Content (%)	Storage Stability (%)
OEA	58.3	58.7	0.4
WEA	57.4	59.5	2.1
WER/PU-CMEA	57.5	58.6	1.1

**Table 6:** TFOT results of different types of emulsified asphalt.

Types of Emulsified Asphalt		Softening Point (°C)	Penetration at 25 °C (0.1 mm)	Softening Point Difference (°C)	Penetration Ratio (%)
OEA	Unaged	46.3	87.2	6.7	58.8
	Aging	53.0	51.3		
WEA	Unaged	58.0	65.6	5.2	62.3
	Aging	63.2	40.9		
WER/PU-CMEA	Unaged	55.5	75.2	4.6	75.7
	Aging	60.1	56.9		

## Data Availability

The original contributions presented in this study are included in the article. Further inquiries can be directed to the corresponding author.
